# Maternal immune activation affects female offspring whisker movements during object exploration in a rat model of neurodevelopmental disorders

**DOI:** 10.1016/j.bbih.2024.100807

**Published:** 2024-06-12

**Authors:** Ugne Simanaviciute, Harry G. Potter, Reinmar Hager, Jocelyn Glazier, Emma Hodson-Tole, John Gigg, Robyn Grant

**Affiliations:** a Faculty of Science and Engineering, Manchester Metropolitan University, Manchester, M1 5GD, UK; b Institute for Behaviour, Sport and Rehabilitation, School of Medicine & Dentistry, University of Central Lancashire, Burnley, BB11 1RA, UK; c Division of Evolution, Infection and Genomics, School of Biological Sciences, Faculty of Biology, Medicine and Health, Manchester Academic Health Science Centre, University of Manchester, Manchester, M13 9PT, UK; d Division of Neuroscience, Faculty of Biology, Medicine and Health, The University of Manchester, Manchester, UK

**Keywords:** Neurodevelopmental disorders, Rat model, Rodent, Behaviour, Vibrissae, Active sensing, Touch, poly(I:C)

## Abstract

Poly I:C rat offspring are used to investigate the effects of *in utero* exposure to maternal immune activation (MIA) and have been suggested as a model of neurodevelopmental disorders (NDD). The behavioural symptoms of this model are diverse and can vary with external factors, including the choice of background strain and husbandry practices. Measuring whisker movements provides quantitative, robust measurements of sensory, motor and cognitive behaviours in rodents. In this study, whisker movements were investigated in 50-day-old male and female offspring of MIA-exposed rat dams and compared to age-matched offspring of control (vehicle) dams. Rat offspring were filmed using high-speed videography in a sequential object exploration task with smooth and textured objects. Poly I:C treatment effects were found in female offspring that did not increase whisker mean angular position during object exploration, especially for the smooth object, indicating an attentional deficit. Whisker tracking during object exploration is demonstrated here, for the first time, as a useful, quick and non-invasive tool to identify both treatment effects and sex differences in a model of MIA-induced NDDs.

## Highlights

• Rat offspring with prenatal MIA from Poly (I:C) show complex and variable behaviours.
• We propose measuring whisker movements to assess subtle, complex behaviours.
• Whisker tracking identified differences between sexes and treatment in MIA offspring.
• E.g. female MIA rat offspring had lower whisker angles during object exploration.
• This suggests an attentional deficit in female offspring of MIA rats.

## 1 Introduction

Neurodevelopmental disorders (NDDs), including schizophrenia, attention deficit hyperactivity disorder and autism spectrum disorder, affect the development of the nervous system and normal brain function. This can have wide-ranging consequences, impacting cognition, emotion, learning, self-control and memory. In pre-clinical studies, maternal immune activation (MIA) rodent models of NDDs have been found to reflect the natural pathogenesis of the NDDs and their symptoms (Woods et al., 2021). A widely used method to induce MIA is gestational exposure to the viral mimetic and Toll-like receptor 3 agonist polyinosinic:polycytidylic acid (Poly I:C, Bucknor et al., 2022). Offspring of Poly I:C dams express behavioural deficits in sensorimotor gating, selective attention, social behaviour, exploratory behaviour, working memory, and cognitive flexibility (Meyer, 2014; Potter et al., 2023). However, the Poly I:C rodent model is challenging, as behavioural findings can vary depending on the rodent genetic background, source and dose of Poly I:C, as well as the gestational timing of treatment (Mueller et al., 2018; Kowash et al., 2019; Murray et al., 2019). Even the type of caging system can affect maternal behaviour and the behaviour of adult offspring (Mueller et al., 2018). Consequently, it is imperative to select a robust and highly quantitative behavioural test to identify such complex behavioural phenotypes.

We posit that measuring whisker movements could be such a robust test. Whiskers are an established sensorimotor model in neuroscience (see Adibi, 2019 for a recent review), and the precise measurement of whisker movements can be captured quickly without any animal training (Simanaviciute et al., 2022), unlike in many other behavioural tasks. Furthermore, measurements are highly granular, including angles and speed. This is in contrast to counts and durations which are more common in behavioural testing, such as the tasks recommended for measuring attention by Lustig et al. (2013). Measuring rodent whisker movements has previously revealed motor, sensory and cognitive deficits in mouse models of neurodegenerative disease (Grant; Garland et al., 2018; Simanaviciute et al., 2020, 2022), although it is probably not possible to disentangle these factors using the current set up. This method has also identified behavioural phenotypes earlier than any other behavioural test, e.g., in a R62 Huntington's Disease mouse model (Garland et al., 2018). Measuring whisker movements during object exploration can identify sensory and attentional deficits (5xFAD mice in Grant et al., 2018; 3xTg-AD mice in Simanaviciute et al., 2022), and reveal sex differences in mouse models of Alzheimer's (Grant et al., 2018) and Huntington's disease (Simanaviciute et al., 2020), which makes it especially aligned for the study of NDDs. Indeed, Lins et al. (2019) specifically recommend investigating sex differences in MIA research, since rat models of NDDs exhibit sex-dependent phenotypes (Snigda et al., 2011; Leger and Neill, 2016; Nikolić et al., 2017; Casquero-Veiga et al., 2023; Potter et al., 2023).

Here, we applied our established mouse whisker measurement protocol (Simanaviciute et al., 2020, 2022) to the rat Poly I:C model to investigate the effects of *in utero* exposure to MIA on whisker movements in offspring at postnatal day (P) 50. We investigated whisker movements before and during object exploration, and examined differences between treatment, sex and object texture. We predict that measures of whisker movements would be sensitive to both treatment and sex effects.

## 2 Materials and methods

### 2.1 Animals

Details of the rat MIA model, including information about animal treatment and housing were reported following the guidelines from Kentner et al. (2019) and can be found in Potter et al. (2023). For our study, 11 dams were pseudo-randomised to treatment group (Excel v2004 random number generator, Microsoft, USA). Dam sample sizes were calculated using the statistical package G*Power v3.1.9.2. Whisker movements of the offspring of these dams were measured once they reached early adulthood, at ∼ P50 (47–53) (Supplementary material A, Table S1) when whisker movements are adult-like (Grant). From a total of 24 MIA offspring rats, 11 were female and 13 male, and from 26 control rats, 14 were female and 12 male. Half of the rats were cross fostered as part of a satellite study; however, this had no significant effect on whisker metrics (Supplementary material B, Tables S2 and S3) nor on other adult behaviours (Potter, 2021). Therefore, data were combined and cross-fostering was not investigated further.

### 2.2 Experimental procedures

All experimental procedures were carried out at the University of Manchester Biological Services Facility, in the light phase of the daily cycle (standard 12h light:dark cycle, lights on at 7:00am). Experiments were performed under Home Office UK project licence (number P473EC3B1) in accordance with the Animals (Scientific Procedures) Act UK 1986. Before being involved in this study, all animals had been exposed to other tasks at P35 (∼15 days before this study, as described in Potter et al., 2023), including novel object recognition (NOR), elevated plus maze and social interaction tasks.

For high-speed filming of whisker movements, a sequential object exploration task was adopted (Supplementary material C, Fig. S1), exact details of which can be found in Landreth et al. (2021). Observers were blind to the rat treatment group throughout the video collection and analysis process. Video clips were selected for analysis based on criteria developed by Grant et al. (2014); that the head was level, the whiskers in view and the rat be travelling toward the object in the pre-contact (PC) section of the clip and the whiskers contacting the object in the during-contact (DC) section of the clip. Clips were tracked using the Automated Rodent Tracker (ARTv2; Gillespie et al., 2019). 2–12 whiskers were detected in each frame (with 5–6 whiskers on each side being usual). 1–4 video clips per rat were included in data analyses (Supplementary material A, Table S1), giving a total of 114 clips, including both PC and DC sections. PC sections ranged from 100 to 291 frames per clip and DC sections ranged from 100 to 459 frames. Whisker metrics included both PC and PC-DC measures of mean angular position (mean whisker angles), whisker amplitude (standard deviation of angular positions multiplied by 2x√2), whisker asymmetry (difference between left and right angular position), whisker spread (mean standard deviation of all tracked whiskers), and retraction and protraction speeds (mean speed of whiskers moving backwards and forwards, respectively). Full definitions for these metrics can be found in Simanaviciute et al. (2020).

### 2.3 Statistical analyses

**Fig. 1:**
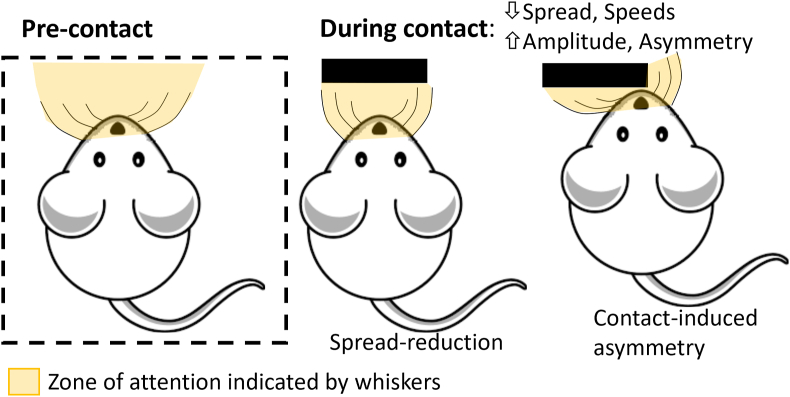
**Summary whisker positional changes observed pre-contact and during contact.** Object contact causes increases in whisker amplitude and asymmetry (termed contact-induced asymmetry) and a reduction in whisker spread and movement speeds (both retraction and protraction speed). The area covered by the whiskers can be thought of as a zone of attention.

Measures of the PC whisker variables were analysed first. Changes in whisker movements during object exploration were analysed by subtracting the DC measures from the PC measures (PC-DC), as per Simanaviciute et al. (2022). Examining PC-DC metrics reveals common contact-related whisker behaviours (Fig. 1), including increasing the number of whisker contacts (increasing protraction angles and decreasing whisker spread during contact), while ensuring light whisker contacts (increasing asymmetry during contact) over a longer period of time (by reducing whisker speeds during contact) (see Grant and Goss, 2022 for a full review).

Linear Mixed-Effects Models (lme4 in R Studio version 1.1.456) were used to analyse the effect of Poly I:C treatment, sex and object texture (or order) on all PC and (PC-DC) whisker variables. The degrees of freedom were approximated using a Kenward-Rodger's method and could fall anywhere between the number of clips (n = 114) and number of individuals (n = 50). A significance value of *p* < 0.05 was used throughout and adjusted in pairwise comparisons with Tukey's method. Data and code used for analysis is referenced in Supplementary material E.

## 3 Results

**Fig. 2:**
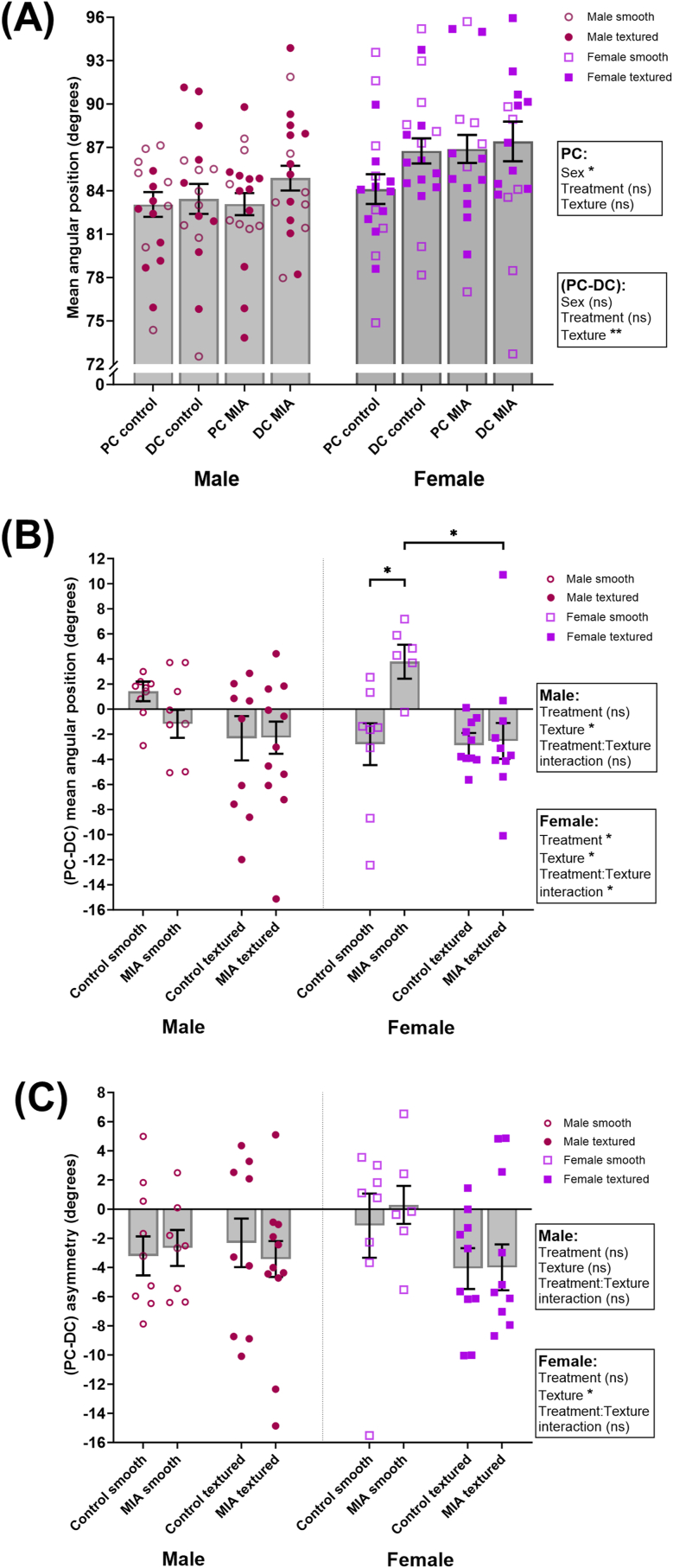
**MIA offspring rat mean angular position (A and B) and asymmetry (C) are affected by treatment and object texture.** A) Significant sex effects were found in PC whisker mean angular position and object texture effects were found in (PC-DC) mean angular position. B) In males, there was a main effect of object texture in (PC-DC) mean angular position. In females, there were effects of treatment, object texture and their interaction in (PC-DC) mean angular position, while pairwise comparisons showed that MIA offspring rat smooth texture group was significantly different to control rats exploring the smooth object and MIA offspring rats exploring the textured object. C) There was a significant effect of object texture in (PC-DC) asymmetry in females, independent of treatment. Males and females were analysed together in A and separately in B and C. The bars indicate the mean values from all the clips (degrees of freedom calculated from a linear mixed-effect model), with error bars representing SEM. Data points show mean values for individual rats, indicated by open circles for male rats investigating smooth object, filled circles for male rats investigating textured object, open squares for female rats investigating smooth object, and filled squares for female rats investigating textured object. PC = pre-contact, DC = during contact, (PC-DC) = contact related changes. Asterisks mark significant values where *p* ≤ 0.05 = *, *p* ≤ 0.01 = ** and n.s. is not significant. Sample sizes: 13 MIA male and 11 MIA female offspring rats, 12 male control and 14 control female rats.

In analyses with males and females combined, both MIA-treated and control rat offspring exhibited the common contact-related behaviours that we have previously observed in mice. During contact with an object, whisker retraction speed, protraction speed and spread were consistently reduced (Supplementary material D, Supplementary Figs. S2C, D, and E), while amplitude and asymmetry increased (Supplementary material D, Supplementary Figs. S2A and B). We next tested for Poly I:C treatment and sex effects. There was no effect of treatment, but a sex effect in PC mean angular position was detected (Fig. 2A, Supplementary material D, Table S4), with female rats having higher PC mean angular positions than males (treatment: F_1, 41.224_ = 2.446, *p* = 0.125 sex: F_1, 41.224_ = 5.240, *p* = 0.027; interaction: F_1, 41.224_ = 1.034, *p* = 0.315). There were no further significant effects of treatment or sex in any of the other whisker variables (all p-values >0.05; Fig. 2, Table S5).

### 3.1 Female offspring

**Fig. 3:**
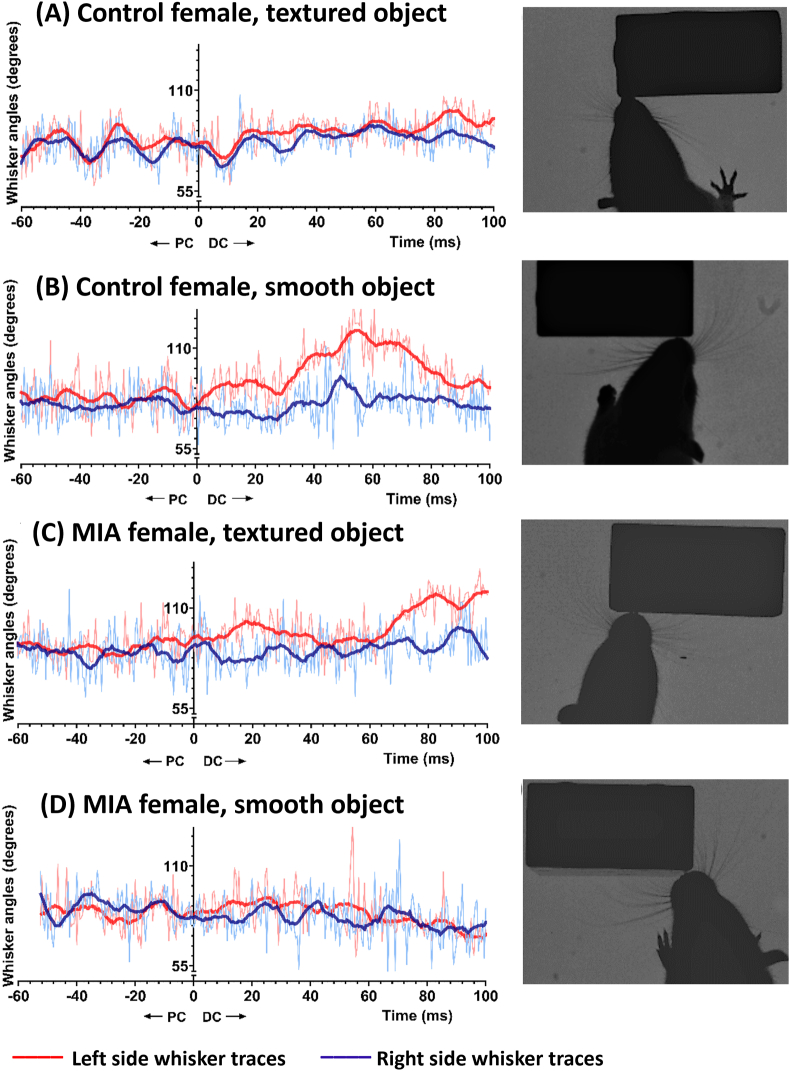
**Example whisker angle traces and video stills of female offspring exploring an object**. Control females (A and C) show predicted object-related whisker behaviours, indicated by asymmetric positioning of the whiskers and high whisker angular positions (e.g., more forward-reaching whiskers) following an object contact. However, the female MIA offspring rat whiskers were more symmetric and less protracted during object contact, especially when contacting the smooth (first) object (B). Whisker traces are shown on the left hand panels. Raw data points are shown in fine lines, and smoothed data (2nd order, 15 neighbours) are presented in thicker lines. Red colour traces are from the whiskers on the left side, and blue from the right side. 0 ms is the point of contact on the x-axis; therefore, left from the Y-axis is pre-contact (PC) and right from the Y-axis is during-contact (DC). Example video clips (one per trace) used here can be found in Supplementary video. Video stills (right hand panel) are selected here when the whiskers are contacting the object at their maximum protraction. (For interpretation of the references to colour in this figure legend, the reader is referred to the Web version of this article.)

Since there was a sex effect, treatment and object texture (or order) was then investigated in males and females separately. (PC-DC) mean angular position in females had a treatment and object texture effect (treatment: F_1, 20.750_ = 5.454, *p* = 0.030; texture: F_1, 51.992_ = 4.573, *p* = 0.037; interaction: F_1, 51.992_ = 4.338, *p* = 0.042, Fig. 2B, Supplementary materials D, Table S6). Post-hoc comparisons showed that female MIA offspring rats contacting the smooth (first) object had significantly higher (PC-DC) mean angular positions compared to female control rats contacting the smooth object (*p* = 0.049) and MIA offspring rats contacting the textured (second) object (*p* = 0.034). This indicates that female MIA offspring rats show the opposite of the predicted contact-related changes in their whisker angular position on the smooth object. This effect can be visualised in the example whisker traces in Fig. 3 (corresponding video clip in Supplementary Video Clip), showing that whiskers of female MIA offspring rats were less protracted during object contact, especially when contacting the smooth (first) object (Fig. 3B).

While there was no treatment effect, a texture (or order) effect was found in (PC-DC) asymmetry in female rats (treatment: F_1, 20.750_ = 0.086, *p* = 0.772; texture: F_1, 51.992_ = 4.274, *p* = 0.044; interaction: F_1, 51.992_ = 0.061, *p* = 0.806, Fig. 2C–Supplementary material D, Table S6). While all other female rats increased their whisker asymmetry during object contact, female MIA offspring rats contacted the smooth object more symmetrically, indicated by positive (PC-DC) values in Fig. 2C and a smaller separation between red and blue traces in Fig. 3B (see also Supplementary Video Clip). There were no further significant effect of treatment nor object texture on any PC whisker metrics, nor other PC-DC metrics (all p-values >0.05).

### 3.2 Male offspring

There was no treatment effect in male rat (PC-DC) mean angular position, but object texture (or order) was significantly different (treatment: F_1, 19.980_ = 0.307, *p* = 0.586; texture: F_1, 53.768_ = 6.856, *p* = 0.012; interaction: F_1, 53.768_ = 1.517, *p* = 0.223, Fig. 2B–Supplementary material D, Table S6). While all other male rats pushed their whiskers more forward during object contact, male control rats on the smooth object reduced their mean angular positions (Fig. 2B). There were no further treatment or object texture (order) effects in male offspring rats in any PC and or (PC-DC) whisker measures (all p-values >0.05).

## 4 Discussion

We investigate here, for the first time, the effect of *in utero* exposure to MIA on whisker movements in P50 offspring, a rat Poly I:C model. When male and female rats were grouped, we saw no effect of Poly I:C treatment on any of the PC or (PC-DC) whisker metrics (Supplementary material D, Supplementary Fig. S2). In agreement, Potter et al. (2023) also found no effect of Poly I:C treatment in adolescent or adult male or female MIA rats in a NOR task used to assess visual learning, and an elevated plus maze task used for measuring anxiety-related behaviour.

However, when we split our data by sex, we found significant treatment effects in female offspring. Sex differences are important to consider in any research and is especially relevant when modelling NDDs, which are expressed differently in males and females (Kokras and Dalla, 2014). We agree with the importance of including both male and female animals in studies and evaluating their neurological symptoms separately, as treatment effects can be masked by innate sex differences. Given the young age of our animals (∼P50), some sex-differences may be due to different developmental trajectories, and we would recommend repeating this study at more ages in order to fully describe their sex differences.

We observed that female MIA offspring rats did not increase their mean angular position during contact with the smooth (first) object, implying they did not engage in the contact-related behaviours that we would usually expect. Conversely, female MIA offspring rats contacting the textured object and control rats touching the smooth object all increased their mean angular position during contact. Both MIA and control female offspring rats contacting the smooth object engaged in contact-induced asymmetry less than male rats (Fig. 2). Positioning whiskers more forward during object contact is associated with focussing of attention (Arkley et al., 2014; Mitchinson et al., 2007, Fig. 1). We would typically expect to see increases in both forward whisker mean angular positions and asymmetry during contact (Berg and Kleinfeld 2003; Grant et al., 2009; Mitchinson et al., 2007). Since these behaviours were absent in the MIA female offspring rats contacting the smooth object, it may suggest abnormal behaviours and attentional deficits in MIA female offspring. We have observed the same behaviour in female 5XFAD mice, a model of Alzheimer's Disease, which was also not present in males (Grant et al., 2020). Potter et al. (2023) also found multiple deficits in attention and problem solving in female MIA offspring rats in more classic behavioural tasks, which further supports our conclusion that attention and executive function are likely to be impacted by the Poly I:C treatment in female rats.

This is the first time we have observed significant treatment effects on whisker movements with different objects. An identical sequential object exploration task was used by Landreth et al. (2021) in a sub-chronic PCP rat model of schizophrenia and did not reveal any differences between the smooth and textured objects. It is worth noting that the objects were very similar and only differed in colour and texture (Supplementary material C, Fig. S1), thus, the novelty of the second object is not especially pronounced. However, the number, type, texture and novelty of objects may affect rodent behaviour in object exploration tasks and should be considered when looking for MIA treatment effects. In our animals, we suggest that treatment and sex differences may primarily manifest during whisker exploration of the first, novel object presented to them. This means that whisker movements could be measured with only one object in the future, further simplifying the set-up for this method. Indeed, we show here that measuring whisker movements offers a quick, non-invasive, quantitative tool that is sensitive enough to identify treatment and sex effects during object exploration in an offspring model of NDDs caused by MIA.

## Funding

This work was supported by the PhD scholarship granted to US by 10.13039/100010014Manchester Metropolitan University. The animal work was funded by a 10.13039/501100000268BBSRC DTP studentship. (BB/M011208/1) granted to HP.

## CRediT authorship contribution statement

**Ugne Simanaviciute:** Data curation, Formal analysis, Investigation, Methodology, Visualization, Writing – original draft, Writing – review & editing. **Harry G. Potter:** Data curation, Investigation, Methodology, Writing – review & editing. **Reinmar Hager:** Methodology, Project administration, Resources, Supervision, Writing – review & editing. **Jocelyn Glazier:** Methodology, Project administration, Resources, Supervision, Writing – review & editing. **Emma Hodson-Tole:** Supervision, Writing – review & editing. **John Gigg:** Conceptualization, Methodology, Project administration, Resources, Supervision, Writing – review & editing. **Robyn Grant:** Conceptualization, Methodology, Project administration, Resources, Software, Supervision, Writing – original draft, Writing – review & editing.

## Declaration of competing interest

We declare no conflicts of interest.

## Data Availability

Data is linked in Supplementary Material. Video clips are available upon request.
