# Supplementary material for: Maternal immune activation affects female offspring whisker movements during object exploration in a rat model of neurodevelopmental disorders
Source: Brain Behav Immun Health. 2024 Jun 12;39:100807. doi: 10.1016/j.bbih.2024.100807 (PMC11233915; doi:10.1016/j.bbih.2024.100807)
Supplement: Multimedia component 2 [file mmc2.docx]

## **Supplementary materials**

### A. Sample sizes

**Supplementary Table S1.** **Number of videos per rat included in analyses.** Sex abbreviated: F – female; M - male. Treatment: Vehicle - injection of 0.9% saline; Poly I:C - injection of polyinosinic:polycytidylic acid to rat dams used in the study. Note that the smooth object was also always the first object, and the textured object was always the second object presented to rats.

| **Rat ID** | **Litter ID** | **Sex** | **Treatment** | **Body weight at PD35 (g)** | **Clips smooth object** | **Clips textured object** |
| --- | --- | --- | --- | --- | --- | --- |
| 551 | Q1 | F | Poly I:C | 136.2 | 0 | 1 |
| 552 | Q4 | F | Poly I:C | 148.3 | 1 | 2 |
| 553 | Q4 | F | Poly I:C | 138.5 | 2 | 1 |
| 554 | Q5 | F | Vehicle | 134.5 | 2 | 2 |
| 601 | Q1 | M | Poly I:C | 133.3 | 0 | 2 |
| 602 | Q2 | M | Vehicle | 166.3 | 0 | 2 |
| 603 | Q5 | M | Vehicle | 167.7 | 4 | 1 |
| 604 | R1 | M | Vehicle | 164.2 | 0 | 1 |
| 431 | R2 | F | Poly I:C | 148.6 | 1 | 1 |
| 555 | R1 | F | Vehicle | 172.3 | 1 | 2 |
| 611 | R2 | M | Poly I:C | 167.4 | 3 | 0 |
| 561 | S1 | F | Vehicle | 133.1 | 0 | 3 |
| 562 | S3 | F | Poly I:C | 125.8 | 0 | 2 |
| 563 | S1 | F | Vehicle | 123.5 | 1 | 0 |
| 612 | R4 | M | Vehicle | 192.0 | 0 | 1 |
| 613 | T2 | M | Poly I:C | 150.6 | 1 | 1 |
| 614 | S1 | M | Vehicle | 148.2 | 1 | 0 |
| 621 | S3 | M | Poly I:C | 152.4 | 0 | 1 |
| 632 | T2 | M | Poly I:C | 168.9 | 2 | 2 |
| 564 | S4 | F | Vehicle | 161.8 | 0 | 2 |
| 565 | S4 | F | Vehicle | 161.2 | 1 | 0 |
| 573 | S4 | F | Vehicle | 139.3 | 0 | 2 |
| 574 | T5 | F | Poly I:C | 148.7 | 1 | 1 |
| 622 | S4 | M | Vehicle | 177.0 | 1 | 2 |
| 623 | T5 | M | Poly I:C | 146.0 | 0 | 2 |
| 633 | S4 | M | Vehicle | 186.7 | 1 | 0 |
| 634 | T5 | M | Poly I:C | 140.9 | 0 | 4 |
| 571 | S5 | F | Vehicle | 147.4 | 1 | 3 |
| 581 | S5 | F | Vehicle | 142.5 | 2 | 0 |
| 624 | S5 | M | Vehicle | 157.8 | 1 | 1 |
| 631 | U2 | M | Poly I:C | 161.0 | 2 | 0 |
| 642 | S5 | M | Vehicle | 158.1 | 2 | 0 |
| 575 | U1 | F | Vehicle | 142.1 | 1 | 2 |
| 582 | U1 | F | Vehicle | 134.2 | 0 | 1 |
| 583 | U3 | F | Poly I:C | 139.8 | 1 | 0 |
| 641 | U3 | M | Poly I:C | 182.5 | 0 | 1 |
| 643 | U1 | M | Vehicle | 153.1 | 1 | 2 |
| 644 | U3 | M | Poly I:C | 149.5 | 3 | 1 |
| 584 | U5 | F | Poly I:C | 138.6 | 0 | 1 |
| 585 | U5 | F | Poly I:C | 131.5 | 0 | 4 |
| 591 | W2 | F | Vehicle | 133.4 | 0 | 1 |
| 592 | X1 | F | Vehicle | 145.4 | 1 | 0 |
| 593 | W5 | F | Poly I:C | 138.6 | 2 | 2 |
| 594 | X1 | F | Vehicle | 139.4 | 0 | 3 |
| 595 | X3 | F | Poly I:C | 153.1 | 0 | 2 |
| 651 | W4 | M | Vehicle | 173.2 | 0 | 1 |
| 652 | X3 | M | Poly I:C | 164.3 | 0 | 1 |
| 653 | W5 | M | Poly I:C | 157.7 | 2 | 1 |
| 654 | X1 | M | Vehicle | 161.7 | 2 | 1 |
| 655 | X3 | M | Poly I:C | 169.9 | 1 | 3 |
|  |  | **Total rats**: 50 | |  | **Total clips**: 114 | |
| **MIA offspring litters** n = 11 (24 rats)  Control litters n = 11 (26 rats) | | **MIA offspring rats**  n = 24 (11 female, 13 male)  **control rats**  n = 26  (14 female, 12 male) | | **Mean weight (g):**  Control female  143.6 ± 3.6;  Control male  167.2 ± 3.8;  Poly I:C female  140.7 ± 2.5;  Poly I:C male  157.3 ± 3.8 | **45 clips** (29 rats) with smooth object | **69 clips** (40 rats) with textured object |

### B. Cross-fostering

**Supplementary Table S2. Summary statistics for cross-fostering effects on pre-contact data.** Linear mixed effect model and pairwise comparisons with Tukey’s adjustment. Asterisks mark significant values where *p* ≤ 0.05 = * and n.s. is not significant. All post-hoc tests were not significant.

| **PC whisker parameters** | **Treatment effect**  **df_1_ = 1, df_2_, F, p** | **Cross-fostering effect**  **df_1_ = 1,**  **df_2_,**  **F,**  **p** | **Treatment: Cross-fostering interaction**  **df_1_ = 1,**  **df_2_,**  **F,**  **p** |
| --- | --- | --- | --- |
| **Amplitude**  **(degrees)** | 44  1.5  0.23 | 44  4.2  **0.047***  - all n.s. in post-hoc tests | 44  0.86  0.36 |
| **Mean angular position**  **(degrees)** | 43  1.4  0.24 | 43  0.028  0.87 | 43  0.21  0.65 |
| **Asymmetry**  **(degrees)** | 44  1.1  0.30 | 44  0.012  0.91 | 44  0.16  0.69 |
| **Retraction speed**  **(degrees/second)** | 41  0.0079  0.93 | 41  0.55  0.46 | 41  1.8  0.19 |
| **Protraction speed**  **(degrees/second)** | 41  0.0046  0.95 | 41  0.091  0.77 | 41  0.29  0.59 |
| **Spread**  **(degrees)** | 41  0.0027  0.96 | 41  2.0  0.17 | 41  0.35  0.56 |

**Supplementary Table 3. Summary statistics for cross-fostering effects on contact-related (PC-DC) data.** Linear mixed effect model and pairwise comparisons with Tukey’s adjustment. All post-hoc tests were not significant.

| **(PC-DC) whisker parameters** | **Treatment effect**  **df_1_ = 1, df_2_, F, p** | **Cross-fostering effect**  **df_1_ = 1,**  **df_2_,**  **F,**  **p** | | **Treatment: Cross-fostering**  **interaction**  **df_1_ = 1,**  **df_2_,**  **F,**  **p** |
| --- | --- | --- | --- | --- |
| **Amplitude**  **(degrees)** | 41  0.033  0.86 | 41  1.5  0.23 | 41  0.22  0.64 | |
| **Mean angular position**  **(degrees)** | 40  0.43  0.52 | 40  0.29  0.59 | 40  0.0047  0.95 | |
| **Asymmetry**  **(degrees)** | 40  0.0047  0.95 | 40  0.35  0.56 | 40  0.019  0.89 | |
| **Retraction speed**  **(degrees/second)** | 40  0.38  0.54 | 40  0.16  0.670 | 40  1.8  0.019 | |
| **Protraction speed**  **(degrees/second)** | 40  1.6  0.22 | 40  1.4  0.25 | 40  1.2  0.28 | |
| **Spread**  **(degrees)** | 42  0.38  0.54 | 42  3.9  0.056 | 42  0.0036  0.95 | |

### C. Experimental Procedures


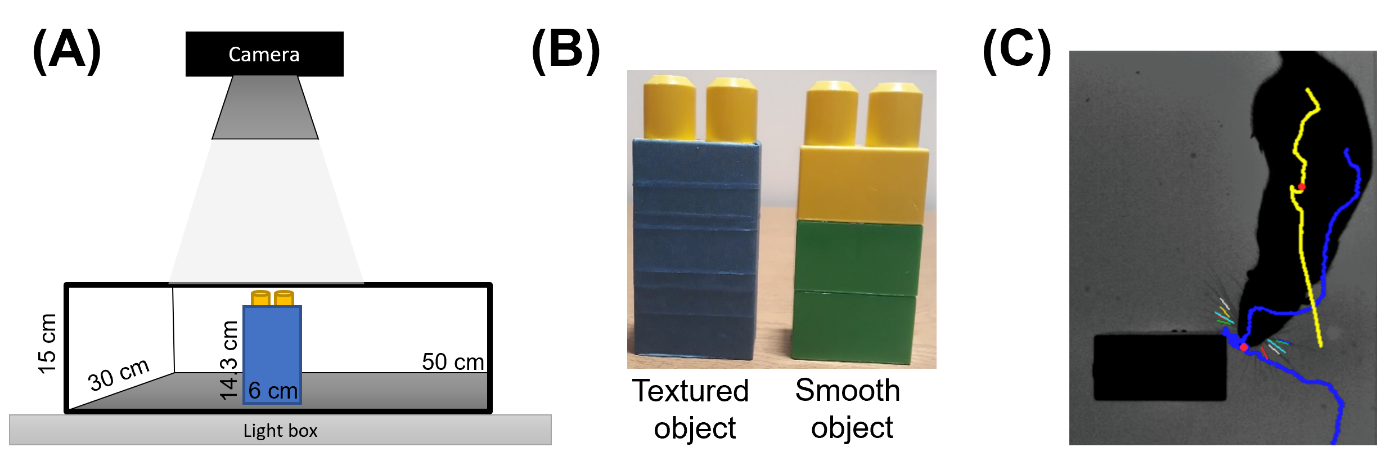


**Supplementary Figure S1. Data collection and video analysis.** A) Filming set-up illustrating the object size and location in relation to the Perspex box, and the distance between the arena and high-speed video camera. B) Smooth and textured plastic bricks that were used as objects in the experiments; C) A video still of an example clip where ARTv2 LocoWhisk software was used to automatically locate the rat centroid and nose point which are marked by the two red points. The paths taken by these two points over the clip duration are shown in yellow (centroid) and blue (nose point) lines. The software also detected whiskers (shown with coloured lines). The field of view shown in A in light grey corresponds to this video still.

### D. Whisker movements

In female rats, there were no differences between treatment groups nor object texture (or order) in PC whisker metrics, including PC amplitude (treatment: F (1, 22.945) = 4.1736, *p* = 0.05271; texture: F (1, 49.191) = 0.0606, *p* = 0.80661; interaction: F (1, 49.191) = 0.0398, *p* = 0.84277), PC mean angular position (treatment: F (1, 22.677) = 2.2889, *p* = 0.1441; texture: F (1, 50.197) = 1.2287, *p* = 0.2729; interaction: F (1, 50.197) = 0.0408, *p* = 0.8408), PC asymmetry (treatment: F (1, 22.368) = 0.1881, *p* = 0.66869; texture: F (1, 50.930) = 2.9567, *p* = 0.09159; interaction: F (1, 50.930) = 0.3150, *p* = 0.57711), PC spread (treatment: F (1, 21.970) = 0.4104, *p* = 0.5284; texture: F (1, 51.504) = 0.1568, *p* = 0.6938; interaction: F (1, 51.504) = 0.2516, *p* = 0.6181), PC retraction speed (treatment: F (1, 20.750) = 0.0251, *p* = 0.8757; texture: F (1, 51.992) = 0.0860, *p* = 0.7705; interaction: F (1, 51.992) = 0.1759, *p* = 0.6767) or PC protraction speed (treatment: F (1, 20.750) = 0.0251, *p* = 0.8757; texture: F (1, 51.992) = 0.0754, *p* = 0.7847; interaction: F (1, 51.992) = 1.3401, *p* = 0.2523). There were no Poly I:C treatment nor object texture (or order) effects in female (PC-DC) whisker amplitude (treatment: F (1, 20.750) = 0.0559, *p* = 0.8154; texture: F (1, 51.992) = 0.6787, *p* = 0.4138; interaction: F (1, 51.992) = 0.0007, *p* = 0.9785), (PC-DC) spread (treatment: F (1, 20.765) = 0.2381, *p* = 0.6307; texture: F (1, 51.994) = 1.3485, *p* = 0.2508; interaction: F (1, 51.994) = 1.8671, *p* = 0.1777), (PC-DC) retraction speed (treatment: F (1, 21.957) = 0.8292, *p* = 0.3724; texture: F (1, 51.518) = 0.8876, *p* = 0.3505; interaction: F (1, 51.518) = 0.1546, *p* = 0.6958) or (PC-DC) protraction speed (treatment: F (1, 20.750) = 1.6326, *p* = 0.2155; texture: F (1, 51.992) = 0.0301, *p* = 0.8630; interaction: F (1, 51.992) = 2.4627, *p* = 0.1226).

There were no treatment or object texture (order) effects in male MIA offspring rats in PC whisker amplitude (treatment: F (1, 19.950) = 0.0293, *p* = 0.86584; texture: F (1, 53.796) = 0.0144, *p* = 0.90503; interaction: F (1, 53.796) = 3.8268, *p* = 0.05564), PC mean angular position (treatment: F (1, 20.305) = 0.3474, *p* = 0.56208; texture: F (1, 53.307) = 3.4287, *p* = 0.06961; interaction: F (1, 53.307) = 0.0843, *p* = 0.77274), PC asymmetry (treatment: F (1, 21.040) = 1.4169, *p* = 0.2472; texture: F (1, 50.573) = 0.0024, *p* = 0.9609; interaction: F (1, 50.573) = 0.6799, *p* = 0.4135), PC spread (treatment: F (1, 17.367) = 0.2893, *p* = 0.5975; texture: F (1, 52.289) = 1.9235, *p* = 0.1714; interaction: F (1, 52.289) = 0.1553, *p* = 0.6952), PC retraction speed (treatment: F (1, 19.337) = 0.5095, *p* = 0.4839; texture: F (1, 53.991) = 0.4689, *p* = 0.4964; interaction: F (1, 53.991) = 0.1304, *p* = 0.7194) or PC protraction speed (treatment: F (1, 18.633) = 0.0006, *p* = 0.9810; texture: F (1, 53.638) = 1.9668, *p* = 0.1666; interaction: F (1, 53.638) = 0.5047, *p* = 0.4805). There were also no treatment effects in male (PC-DC) whisker amplitude (treatment: F (1, 18.861) < 0.0001, *p* = 0.9996; texture: F (1, 53.798) = 0.4104, *p* = 0.5245; interaction: F (1, 53.798) = 1.0143, *p* = 0.3184), (PC-DC) asymmetry (Figure 2C, Supplementary Table 6), (PC-DC) spread (treatment: F (1, 20.635) = 0.0303, *p* = 0.8636; texture: F (1, 52.454) = 0.9496, *p* = 0.3343; interaction: F (1, 52.454) = 0.2028, *p* = 0.6543), (PC-DC) retraction speed (treatment: F (1, 17.367) = 0.0625, *p* = 0.8056; texture: F (1, 52.289) = 0.2397, *p* = 0.6265; interaction: F (1, 52.289) = 0.0011, *p* = 0.9740) or (PC-DC) protraction speed (treatment: F (1, 17.367) = 1.1098, *p* = 0.3066; texture: F (1, 52.289) = 0.1330, *p* = 0.7169; interaction: F (1, 52.289) = 0.0115, *p* = 0.9149).


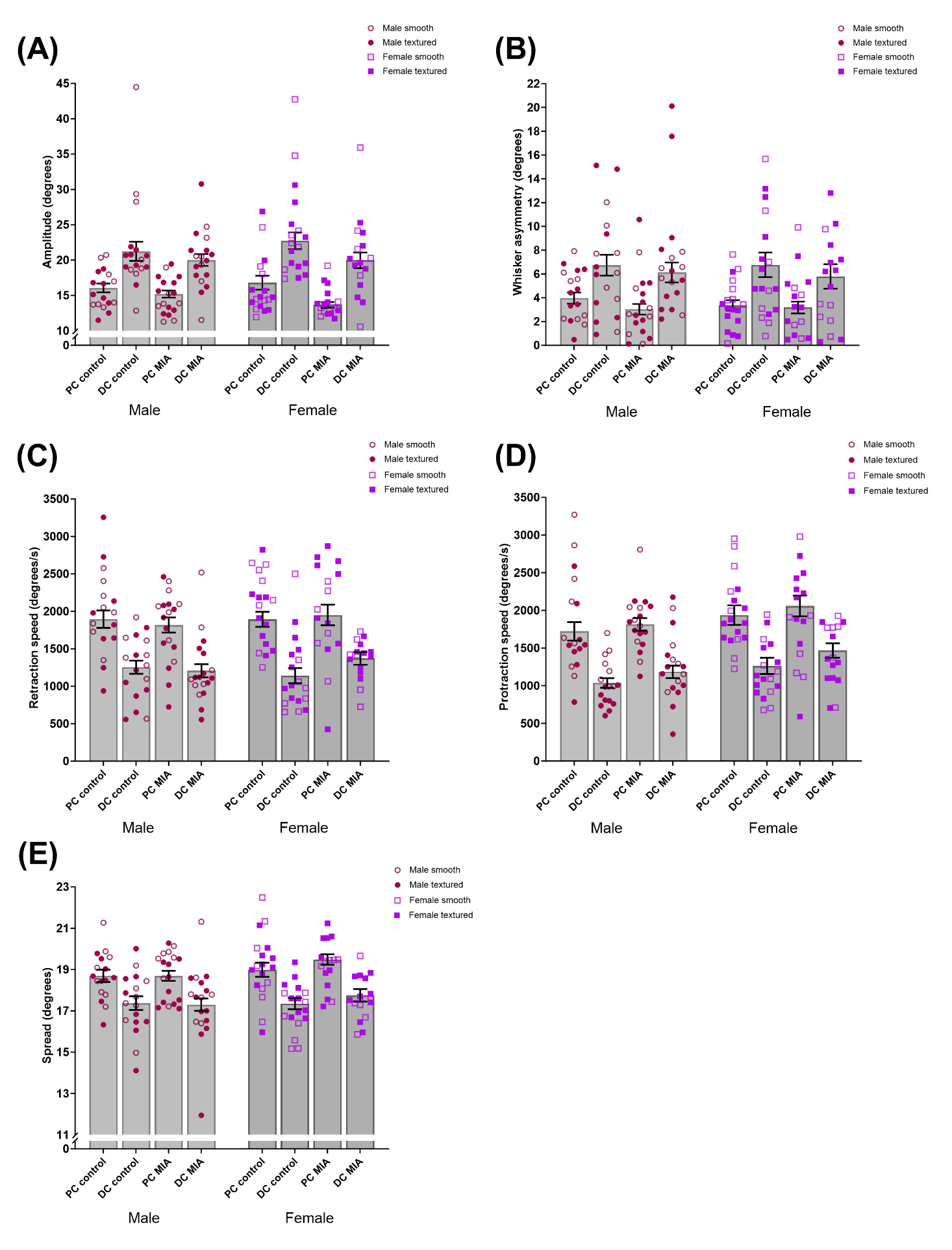


**Supplementary Figure S2.** **Poly I:C treatment, sex and object texture (or order)** **did not have significant effects on whisker amplitude, asymmetry, protraction speed, retraction speed or spread**, when analysed with male and female rats combined. The bars indicate the mean values from all the clips (degrees of freedom calculated from a linear mixed-effect model), with bars representing SEM. Data points show mean values for individual rats, indicated by open circles for male rats investigating smooth (first) object, filled circles for male rats investigating textured (second) object, open squares for female rats investigating smooth (first) object, and filled squares for female rats investigating textured (second) object. PC = pre-contact, DC = during contact.

**Supplementary Table S4. Summary statistics for pre-contact data.** Linear mixed effect model and pairwise comparisons with Tukey’s adjustment. Treatment, Sex and Treatment:Sex interaction columns are reported from Treatment*Sex tests, and Texture column is reported from Treatment*Texture tests. Asterisks mark significant values where *p* ≤ 0.05 = **.*

| **PC whisker parameters** | **Treatment effect**  **df_1_ = 1, df_2_, F, p** | **Sex effect**  **df_1_ = 1,**  **df_2_,**  **F,**  **p** | **Treatment:Sex**  **interaction**  **df_1_ = 1,**  **df_2_,**  **F,**  **p** | **Texture (order) effect**  **df_1_ = 1,**  **df_2_,**  **F,**  **p** |
| --- | --- | --- | --- | --- |
| **Amplitude (degrees)** | 43  2.2  0.15 | 43  0.74  0.39 | 43  1.8  0.19 | 100  0.29  0.59 |
| **Mean angular position**  **(degrees)** | 41  2.5  0.13 | 41  5.2  **0.027*** | 41  1.0  0.32 | 110  3.6  0.059 |
| **Asymmetry**  **(degrees)** | 43  1.4  0.25 | 43  0.75  0.39 | 43  0.74  0.40 | 110  2.0  0.16 |
| **Retraction speed**  **(degrees/second)** | 38  0.098  0.76 | 38  0.31  0.58 | 38  0.63  0.43 | 110  0.62  0.43 |
| **Protraction speed**  **(degrees/second)** | 36  0.19  0.67 | 36  3.3  0.076 | 36  0.31  0.58 | 110  0.88  0.35 |
| **Spread**  **(degrees)** | 36  0.14  0.71 | 36  2.5  0.12 | 36  1.8  0.19 | 110  0.40  0.53 |

**Supplementary Table S5. Summary statistics for contact-related (PC-DC) data.** Linear mixed effect model and pairwise comparisons with Tukey’s adjustment. Treatment, Sex and Treatment:Sex interaction columns are reported from Treatment*Sex tests, and Texture column is reported from Treatment*Texture tests. Asterisks mark significant values where *p* ≤ 0.01 = **.

| **(PC-DC) whisker parameters** | **Treatment effect**  **df_1_ = 1, df_2_, F, p** | **Sex effect**  **df_1_ = 1,**  **df_2_,**  **F,**  **p** | **Treatment:Sex interaction**  **df_1_ = 1,**  **df_2_,**  **F,**  **p** | **Texture (order) effect**  **df_1_ = 1,**  **df_2_,**  **F,**  **p** |
| --- | --- | --- | --- | --- |
| **Amplitude**  **(degrees)** | 38  0.0036  0.95 | 38  0.72  0.40 | 38  0.065  0.80 | 110  1.1  0.31 |
| **Mean angular position**  **(degrees)** | 36  0.42  0.52 | 36  0.38  0.54 | 36  2.9  0.10 | 110  7.1  **0.0090**** |
| **Asymmetry**  **(degrees)** | 36  0.017  0.90 | 36  0.042  0.84 | 36  0.22  0.64 | 110  1.9  0.18 |
| **Retraction speed**  **(degrees/second)** | 36  0.66  0.42 | 36  0.11  0.75 | 36  0.29  0.59 | 110  0.055  0.82 |
| **Protraction speed**  **(degrees/second)** | 36  1.6  0.22 | 36  0.0012  0.97 | 36  0.00050  0.98 | 110  0.024  0.88 |
| **Spread**  **(degrees)** | 41  0.059  0.81 | 41  0.59  0.45 | 41  0.0049  0.94 | 110  2.2  0.14 |

**Supplementary Table S6. Significant whisker parameters** in detail. Here, female and male MIA offspring rats were analysed separately. Linear mixed effect model and pairwise comparisons with Tukey’s adjustment. Asterisks mark significant values where *p* ≤ 0.05 = *.

|  | | | | **Post-hoc** | | | |
| --- | --- | --- | --- | --- | --- | --- | --- |
| **Whisker parameters** | **Treatment effect**  **df_1_ = 1,**  **df_2_,**  **F,**  **p** | **Texture (order) effect**    **df_1_ = 1,**  **df_2_,**  **F,**  **p** | **Treatment:**  **Texture (order) interaction**  **df_1_ = 1,**  **df_2_,**  **F,**  **p** | MIA vs control offspring  **smooth (first)**  df_1_,  t-ratio,  p | MIA vs control  Offspring  **textured (second)**  df_1_,  t-ratio,  p | smooth (first) vs textured (second)  **MIA offspring**  df_1_,  t-ratio,  p | smooth (first) vs textured (second)  **control**  **offspring**  df_1_,  t-ratio,  p |
| **Male**  **(PC-DC) mean angular position**  **(degrees)** | 20  0.31  0.59 | 54  6.9  **0.012*** | 54  1.5  0.22 | 33  -1.2  0.64 | 37  0.39  0.98 | 54  1.1  0.70 | 53  2.5  0.073 |
| **Female**  **(PC-DC) mean angular position**  **(degrees)** | 21  5.5  **0.030*** | 52  4.6  **0.037*** | 52  4.3  **0.042*** | 44  2.7  **0.049*** | 25  0.20  1.0 | 52  2.8  **0.034*** | 52  0.042  1.0 |
| **Male**  **(PC-DC) asymmetry**  **(degrees)** | 17  0.17  0.69 | 52  0.014  0.91 | 52  0.22  0.64 | 26  0.045  1.0 | 41  -0.64  0.92 | 48  0.29  0.99 | 54  -0.38  0.98 |
| **Female**  **(PC-DC) asymmetry**  **(degrees)** | 21  0.086  0.77 | 52  4.3  **0.044*** | 52  0.061  0.81 | 44  0.33  0.99 | 25  0.038  1.0 | 52  1.5  0.42 | 52  1.4  0.52 |

**Supplementary Table S7. Summary statistics for Treatment*Texture effects.** Linear mixed effect model and pairwise comparisons with Tukey’s adjustment. Texture effect is reported in main text Tables 2 and 3. Males and females analysed together.

| **Whisker parameters** | **Treatment effect**  **df_1_ = 1, df_2_, F, p** | **Treatment:Texture**  **interaction**  **df_1_ = 1,**  **df_2_,**  **F,**  **p** |
| --- | --- | --- |
| **PC amplitude (degrees)** | 46  2.5  0.13 | 100  3.0  0.090 |
| **PC mean angular position (degrees)** | 45  1.4  0.24 | 110  0.070  0.79 |
| **PC asymmetry (degrees)** | 44  1.3  0.26 | 110  0.20  0.65 |
| **PC retraction speed (degrees/second)** | 39  0.14  0.71 | 110  0.021  0.89 |
| **PC protraction speed (degrees/second)** | 38  0.010  0.92 | 110  1.4  0.24 |
| **PC spread (degrees)** | 40  0.0035  0.95 | 110  0.015  0.90 |
| **(PC-DC) amplitude (degrees)** | 40  0.0042  0.95 | 110  0.35  0.56 |
| **(PC-DC) mean angular position (degrees)** | 38  0.82  0.37 | 110  0.047  0.83 |
| **(PC-DC) asymmetry (degrees)** | 38  0.0044  0.95 | 110  0.058  0.81 |
| **(PC-DC) retraction speed (degrees/second)** | 38  0.83  0.37 | 110  0.080  0.78 |
| **(PC-DC) protraction speed (degrees/second)** | 38  2.3  0.14 | 110  1.4  0.24 |
| **(PC-DC) spread (degrees)** | 43  0.32  0.58 | 110  1.9  0.17 |

### E. Data analysis

<https://github.com/usimana/MIA_rats>
